# Dynamic of plasmodium falciparum chloroquine resistance transporter gene Pfcrt K76T mutation five years after withdrawal of chloroquine in Burkina Faso

**DOI:** 10.11604/pamj.2015.21.101.6437

**Published:** 2015-06-09

**Authors:** Paul Sondo, Karim Derra, Zekiba Tarnagda, Seydou Diallo Nakanabo, Odile Zampa, Adama Kazienga, Innocent Valea, Hermann Sorgho, Jean-Bosco Ouedraogo, Tinga Robert Guiguemde, Halidou Tinto

**Affiliations:** 1 IRSS, Clinical Research Unit of Nanoro, CMA Saint Camille de Nanoro, Ouagadougou CMS 11, Burkina Faso; 2 Center Muraz of Bobo-Dioulasso, Burkina Faso

**Keywords:** Malaria, plasmodium falciparum, chloroquine resistance, pfcrt

## Abstract

We investigated the evolution of Pfcrt K76T mutation five years after the withdrawal of chloroquine in Burkina Faso. A total of 675 clinical isolates collected from October 2010 to September 2012 were successfully genotyped. Single nucleotide polymorphism in Pfcrt (codon 76) gene was analyzed. The prevalence of resistant Pfcrt 76T allele was 20.55%. There was a progressive decrease of the proportion of mutant type pfcrt T76 from 2010 to 2012 (X2=5.508 p=0.0189). Our results suggest a progressive return of the wild type Pfcrt K76 in Burkina Faso but the prevalence of the mutants Pfcrt T76 still remains high.

## Introduction

Like several malaria endemic countries, Artesunate-Amodiaquine (ASAQ) and Artemether-Lumefantrine (AL) are the two combinations recommended in Burkina Faso for the treatment of uncomplicated malaria since the change of the national policy in 2005. Nevertheless recently, there were reports on the decreasing sensitivity of ACTs recorded along the Thailand and Myanmar border [1]. More recently a study conducted in Burkina Faso showed a high Lumefantine inhibition concentration (IC50) five years after the policy change, suggesting a decreasing efficacy of one of the two artemisinin based combination therapies (ACTs) recommended in the country [2]. In such context the development of alternative therapies is needed to anticipate the worldwide expansion of the ACTs resistance reported in Asia. That includes the use of old drugs such as chloroquine (CQ) in combination with partner drugs. Indeed the combination Chloroquine-Azithomycin has showed promising results in pregnant women [3, 4]. This is particularly important in a context where few studies particularly in Malawi and Kenya showed that CQ resistance (CQ-R) decreases few years after its withdrawn and its replacement by ACTs [5–7]. These findings raise the issue of the reintroduction of chloroquine in the panel of potential alternative therapies face to ACTs resistance in the future. In such context there is a need of investigating the evolution of CQ-R to document its sensitivity profile. However currently there is an ethical issue of testing CQ in vivo in patients while a good efficacy of ACTs is still reported [8]. The only existing tools to overcome this problem is either the ex vivo test or testing the CQ-R molecular makers and particularly the Pfcrt gene [9]. However, due to technical constraints of ex vivo tests, the latter seems to be the more appropriate tool. Thus our aim in this study was to investigate the prevalence of Pfcrt K76T mutation five years after the policy change in Burkina Faso.

## Methods

The study was carried out from October 2010 to September 2012 at two peripheral health facilities (Nanoro and Nazoanga) of the Nanoro health district (NHD) in Burkina Faso. This is part of a study entitled: “Pharmacovigilance for artemisinin-based combination treatments in Africa” which had one component aiming at assessing the effectiveness of artesunate-amodiaquine (Arsucam^®^) versus artemether-lumefantrine (Coartem^®^) with a molecular analysis of resistance markers nested to it. Details of the study methodology have been described in detail elsewhere (ClinicalTrials.gov Identifier: NCT01232530). For the current analysis, clinical isolates from day 0 were genotyped. DNA was extracted from dried blood spots using Qiagen Kit and restriction fragment length polymorphism was used to detect the Pfcrt K76T mutation. Data were analyzed using STATA 8 (Stata Corp. 2003). A Chi-square test was used to assess the temporal fluctuation of the proportions. A value of P < 0.05 was considered as statistically significant. The study was approved by Center Muraz Institutional Ethics Committee, Burkina Faso National Ethics Committee.

## Results

**Figure 1:**
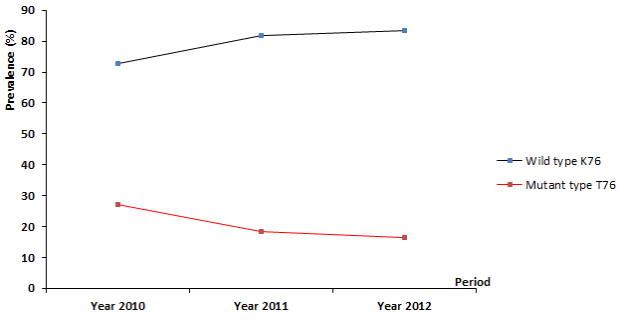
Temporal evolution of Pfcrt (codon 76) genotypes profiles; the figure 1 represents the temporal evolution of Pfcrt codon 76 genotypes profiles from 2010 to 2012. The figure shows a progressive decrease of the mutants Pfcrt T76 (in red) whilst an increase of the wild type PfcrtK76 (in blue) is observed

Overall, 675 blood samples were genotyped. Ninety one samples (13.48%) carrying both wild and mutant Pfcrtalleles and for which related frequencies could not be determined were excluded. The prevalence of wild type PfcrtK76 was 79.45% (464/584). The proportion of mutant type Pfcrt T76 was 20.55% (120/584). The temporal evolution of Pfcrt codon 76 genotype profiles showed a decrease of the prevalence of the mutant type while those of the wild type increased progressively (Figure 1). From 2010 to 2012, the prevalence of the mutants Pfcrt T76 dropped from 27.22% (46/169) to 16.49% (16/97) while the prevalence of the wild type PfcrtK76 increased from 72.78% (123/169) to 83.51% (81/97) showing then a resurgence of CQ sensitive P. falciparumisolates in Nanoro area (X2=5.508 p=0.0189).

## Discussion

Our study aimed at assessing if parasites resistance profile has been affected by the implementation of the new treatment policies. Before the policy change, several studies have reported a high level of mutants Pfcrt T76 allele up to 65% in the country [10]. This study shows a decrease of this mutation five years after the withdrawal of chloroquine. Indeed, the prevalence reported in our study represents about half of that reported five years earlier and confirms the decrease of ex vivo CQ-R rate reported by Tinto et al in Bobo-Dioulasso at the same period [2]. These findings suggest that CQ-R may be decreasing following the implementation of the new anti-malarial drug policy using ACTs in Burkina Faso. Similar findings have been reported in many African countries and particularly in Malawi where, no CQ-R was found nine years after the with drawn of CQ [5, 6]. Nevertheless, the proportion reported in our study is still high compared to that reported in Malawi. This difference can be explained by several factors including the short duration between the introduction of the new treatment policy and our study which occurred only five years later when in Malawi it was nine years. In addition the use of Amodiaquine as partner drug in the combination ASAQ that is widely used in the country could lead to a selective pressureof Pfcrt T76mutant allele due to its similar chemical structure with chloroquine. Indeed the positive correlation between ex vivo IC50 values of CQ and amodiaquine reported by several authors and more recently by Tinto et al in Bobo-Dioulasso at the same period indicates cross-resistance and may also explain the still high prevalence of the mutant allele found in this study [2].

## Conclusion

There is a progressive return of the wild type Pfcrt K76 in Burkina Faso but the prevalence of the mutants Pfcrt T76 is still high. Considering that, the possible reintroduction of CQ in the potential panel of alternative treatments in Burkina Faso could not actually be recommended.
